# CDK5 interacts with MST2 and modulates the Hippo signalling pathway

**DOI:** 10.1002/2211-5463.13962

**Published:** 2024-12-30

**Authors:** Mehak Passi, Jan B. Stöckl, Thomas Fröhlich, Simone Moser, Angelika M. Vollmar, Stefan Zahler

**Affiliations:** ^1^ Center for Drug Research Ludwig‐Maximilians‐University Munich Germany; ^2^ Laboratory for Functional Genome Analysis, Gene Center Munich Ludwig‐Maximilians‐University Munich Germany; ^3^ Institute of Pharmacy University of Innsbruck Austria

**Keywords:** CDK5, Hippo, TAZ, YAP, yeast‐two‐hybrid

## Abstract

MST2 (STK3) is a major upstream kinase in the Hippo signalling pathway, an evolutionary conserved pathway in regulation of organ size, self‐renewal and tissue homeostasis. Its downstream effectors are the transcriptional regulators YAP and TAZ. This pathway is regulated by a variety of factors, such as substrate stiffness or cell–cell contacts. Using a yeast two‐hybrid screen, we detected a novel interaction between the kinases MST2 and CDK5, which we further confirmed by co‐immunoprecipitation experiments. Cyclin‐dependent kinase 5 (CDK5) is an unusual member of the family of cyclin‐dependent kinases, involved in tumour growth and angiogenesis. Although a link between CDK5 and Hippo has been previously postulated, the mode of action is still elusive. Here, we show that knockdown of CDK5 causes reduced transcriptional activity of YAP and that CDK5 influences the phosphorylation levels of the Hippo upstream kinase LATS1. Moreover, a phosphoproteomics approach revealed that CDK5 interferes with the phosphorylation of DLG5, another upstream kinase, which regulates the Hippo pathway. Hence, CDK5 seems to act as a signalling hub for integrating the Hippo pathway and other signalling cascades. These interactions might have important implications for the use of CDK5 inhibitors, which are already in clinical use for tumour diseases.

## Graphical abstract

By using a yeast‐two‐hybrid system, immune precipitation and a proteomics approach, we identify a novel interaction between CDK5 and MST2, which modulates the mechano‐sensitive Hippo signalling pathway in a liver tumour cell line.

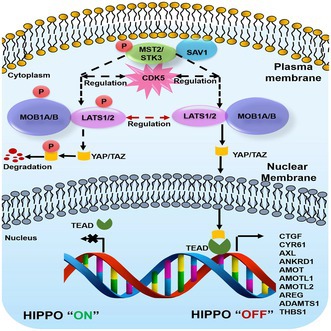

**Abbreviations**
BSA: bovine serum albumin
CDK5: cyclin‐dependent kinase 5
CTGF: connective tissue growth factor
Cyr61: cysteine‐rich angiogenic inducer 61
DLG5: disks large homologue 5
FCS: fetal calf serum
LATS: large tumour suppressor kinase
MAPK: mitogen‐activated protein kinase
MST2: mammalian sterile‐20‐like (MST) kinase 2
RIPA: radioimmunoprecipitation assay buffer
STK3: serine/threonine‐protein kinase 3
TAZ: transcriptional co‐activator with PDZ‐binding motif
TEAD: TEA domain family member
YAP: YES‐associated protein

Cyclin‐dependent kinase 5 (CDK5) is an atypical cyclin‐dependent kinase, which has initially been described in the central nervous system [1]. Binding to its noncyclin activators p35, p39, p25 and p29 regulates the enzymatic activity of CDK5 [2]. Apart from its role in the central nervous system (CNS), CDK5 has been implicated in various types of cancers such as breast, prostate, HCC, breast, lung, ovarian and thyroid [3]. CDK5 has a large number of different substrates and modulates various signalling pathways, such as Notch, Rho GTPase and MAPK, which, in turn, regulate the cytoskeleton, cell adhesion and putatively metastasis [2]. CDK5 also acts as a crucial regulator of tumour angiogenesis by contributing to endothelial cell survival and migration [4, 5, 6].

Due to its multiple roles, we hypothesised that CDK5 has further, still un‐identified protein interaction partners. Therefore, we performed an unbiased screening to identify further protein–protein interactions of CDK5. Yeast two‐hybrid systems are valuable tools for the detection of protein–protein interactions in living yeast cells. Using CDK5 as bait, we identified the kinase MST2 (also known as STK3), as a binding partner.

MST2 is a key player of the Hippo signalling pathway, which plays an important role in the regulation of tissue growth, stem cell activity and tissue architecture. It acts as the main signalling hub for the integration of various cellular inputs, such as cell–cell contact, cell density, cell shape, mechanotransduction, cell polarity and tissue architecture in order to regulate cell growth [7]. Mainly driven by the transcriptional activity of the core kinases MST1/2 and LATS1/2, the Hippo pathway regulates the activity of the oncoproteins YAP/TAZ through a cascade of phosphorylation events, caused and modulated by various regulatory feedback mechanisms occurring within the tissues. In general, when Hippo signalling is active, MST1/2 kinases undergo auto‐activation through auto‐phosphorylation on the activation loop of the MST dimer MST1 at Thr183 and MST2 at Thr180. Further, the MST1 and MST2 kinases activate the LATS1 and LATS2 kinases by phosphorylation at Thr1079 and Thr1041, respectively, and by interacting with MOB1A/MOB1B. The activated LATS kinases phosphorylate YAP and TAZ and lead to its cytoplasmic sequestration [8, 9]. When LATS kinases are inactive and the Hippo pathway is switched off, YAP/TAZ are not phosphorylated and are then translocated to the nucleus. There they bind to members of the TEAD transcription factor family (TEAD1‐4) to mediate target gene expression such as connective tissue growth factor (CTGF), cysteine‐rich angiogenic inducer 61 (Cyr 61) and others to promote cell growth, proliferation, migration and survival. Dysregulation of the Hippo pathway may result in uncontrolled cell proliferation and tissue growth because of increased activity of YAP/TAZ [10, 11, 12, 13, 14].

In this study, we demonstrate that CDK5 interacts with MST2 and influences the Hippo pathway by regulating the activity of the oncoproteins YAP/TAZ. We further reveal how CDK5 influences the phosphorylation levels of kinases involved in Hippo pathway. We also examine the changes in the phosphoproteome occurring due to knockdown of CDK5, and how CDK5 interacts with other pathways.

## Materials and methods

### Antibodies

The following primary antibodies were used for immunostaining and western blot: rabbit anti‐YAP (D8H1, Cell Signaling Technology, Danvers, MA, USA), rabbit anti‐phospho YAP (Ser127, D9W21, Cell Signaling Technology), rabbit anti‐LATS1 (C66B5, Cell Signaling Technology), rabbit anti‐phosphor LATS (Thr1079, Cell Signaling Technology), rabbit anti‐phosphor LATS1 (Ser909, Cell Signaling Technology), rabbit anti‐TAZ (E8E9G, Cell Signaling Technology), rabbit anti‐phosphor Taz (Ser89, Cell Signaling Technology), LATS2 (D83D6, Cell Signaling Technology), rabbit anti‐STK3/MST2 (EP1466Y, Abcam, Cambridge, UK), rabbit anti‐phospho MST1/MST2 (Thr183/Thr180, Cell Signaling Technology) and mouse anti‐CDK5 (DC34, Invitrogen, Darmstadt, Germany).

Goat anti‐rabbit IgG Alexa Fluor 488 (Life Technologies, Darmstadt, Germany) was used as secondary antibody for immunostaining. Goat anti‐mouse IgG HRP conjugate (Cell signalling) and Goat anti‐rabbit HRP conjugate (Dianova, Hamburg, Germany) were used as secondary antibodies for western blot.

### Yeast two hybrid

Cloning Procedure: CDK5 was amplified from a CDK5 containing plasmid for mammalian expression (RRID: Addgene_1870) using the following primers adding BamHI and EcoRI restriction sites:

Forward primer: 5′‐C ATG GAG GCC GAATTC ATG CAG AAA TAC GAG AAA CTG GAA‐3′.

Reverse primer: 5′‐GC AGGTCGACGGATCC GGA TCC GGG CGG ACA GAA GTC GGA‐3′.

The PCR products were analysed with a 1% agarose gel and purified using a gel extraction kit (Qiagen, Venlo, Netherlands). The purified insert and the bait vector pGBKT7 (Takara Bio, San Jose, CA, USA) were digested with BamHI and EcoRI (Thermo Fisher Scientific, Darmstadt, Germany); lower concentrations than indicated in the protocol were used for EcoRI and CDK5, since CDK5 contains a restriction site for EcoRI. The vector was further dephosphorylated by directly adding 1 μL of FastAP Thermosensitive Alkaline Phosphatase (Thermo Scientific, Darmstadt, Germany). Vector and insert were again purified using a gel extraction kit. Ligation was carried out in a 1 : 3 ratio (vector : insert) using the protocol from Thermo Fisher Scientific at room temperature for 30 min, followed by heat shock transformation of the ligation mixture into chemical competent *Escherichia coli* DH5‐α. Plasmid isolation was performed using the QIAprep® Spin Miniprep Kit (Qiagen). Plasmids were analysed by PCR and sequencing using a standard T7 primer (Eurofins, Munich, Germany).

The Matchmaker Gold yeast two‐hybrid system (Takara Bio) was used to screen for interaction partners of CDK5 following the manufacturer's instructions. In brief, CDK5 was amplified from pCMV neo with primers adding restriction sites for BamHI and EcoRI and subsequently cloned into the pGBKT7 bait vector. The vector was introduced into the Y2H Gold reporter strain, which was then mated with the Mate&Plate™ library—Universal Human (normalised) (Takara Bio) in yeast strain Y187. The mating efficiency was calculated as 10.1% by plating serial dilutions. The screening was performed following the Matchmaker manual. An aliquot of 4.7 × 10^7^ cfu was screened (15‐fold library coverage) on four plates (15 cm diameter) containing DDO/X/A medium. After incubation, 316 blue colonies were picked from the four plates and respotted on DDO/X/A and QDO/X/A plates, respectively. Colonies which showed robust growth and blue colour on both plates were classified as ‘hits’, and colony PCR was performed for those colonies. Samples showing a band on an agarose gel were analysed by sequencing (152 samples, Eurofins Genomics, T7 standard sequencing primer) MST2 was identified 36 times. One colony containing the MST2 hit was selected, and the plasmid was isolated (Easy Yeast Plasmid Isolation Kit, Clontech, San Jose, CA, USA), amplified in *E. coli* and retransformed into the Y2H Gold reporter strain already containing the CDK5 bait plasmid. As control, the reporter strain containing the CDK5 bait plasmid was transformed with an empty bait vector not carrying any insert. A prey rescue experiment was performed confirming the interaction between CDK5 and MST2.

### Creation of Huh7 nt and Huh7 CDK5 KD cell lines

For the creation of Huh7 nt cells and Huh7 CDK5 KD cells, transduction of Huh7 cells (obtained from the Japanese Collection of Research Bioresources JCRB) was carried out with Cdk5 shRNA and nt shRNA Cdk5 MISSION® shRNA Lentiviral Transduction Particles (Vector: pLKO.1‐puro; SHCLNVNM_ 004935; Clone ID: (a) TRCN0000021465, (b) TRCN0000021466, (c) TRCN0000021467, (d) TRCN0000194974, (e) TRCN0000195513; Sigma‐Aldrich, Taufkirchen, Germany) and MISSION® pLKO.1‐puro Non‐Mammalian shRNA Control Transduction Particles (SHC002V; Sigma‐Aldrich) according to the manufacturer's protocol. Both cell lines were transduced with a multiplicity of infection (MOI) of one and successfully transduced cells were selected by adding 2 μg·mL^−1^ puromycin to the medium. Puromycin concentration was reduced to 1 μg·mL^−1^ after initial selection to ensure stable transfection with CDK5 shRNA and ntRNA. After the initial selection, puromycin concentration was reduced to 1 μg·mL^−1^ for further cultivation to ensure stable transfection with Cdk5 and nt shRNA. The most efficient and well‐tolerated clones were selected through western blot analysis, as previously described [15].

Successfully transduced Huh7 nt and Huh7 CDK5 KD cells were cultured with high glucose DMEM supplemented with 10% FCS (PAN Biotech GmbH, Aidenbach, Germany) and Puromycin (50 μL·mL^−1^) and cultivated at 37° under 5% CO_2_ atmosphere. Before passing and seeding of cells, all the culture flasks, multiwall plates and dishes were coated with collagen G (0.001% in PBS, Sigma‐Aldrich, Taufkirchen, Germany). Efficiency of the knockdown is about 70% reduction versus nontargeting control as assessed by western blot (Fig. S1).

### Transfections and plasmids

Luciferase reporter construct pGL4.74 (renilla control) and the SRF reporter construct pGL4.34 [luc2P/SRF‐RE/Hygro were purchased from Promega (Madison, WI, USA)]. The 8xGTIIC‐luciferase was a gift from Stefano Piccolo (Addgene, Watertown, MA, USA plasmid #34615; http://n2t.net/addgene:34615; RRID:Addgene_34615). This synthetic TEAD reporter has previously been described as reporter for YAP/TAZ activity [16]. Huh7 nt cells were transfected using Lipofectamine 3000 transfection kit (Thermo Fisher) according to manufacturer instructions. For reporter gene assay, Fugene transfection reagent (Promega) was used to perform transfection according to the manufacturer's protocol. Western blot and immunostaining were performed between 24 and 48 h after transfection.

### Luciferase reporter gene assays

An Orion II microplate luminometer equipped with simplicity Software (Berthold Detection Systems GmbH, Bad Wildbad, Germany) was used to perform Luciferase reporter gene assays. Initially, cells (seeding density of 1 × 10E6 cells per well) were transfected with YAP and Renilla Luciferase reporters using Fugene transfection reagent for 24 h in 6 well plates. Later the next day, cells were reseeded in 24 well plates and stimulated with thrombin for 1 h. Firefly and Renilla vectors were used in a ratio of 10 : 1. Finally, Firefly/Renilla luciferase intensity levels were determined with the Dual‐Luciferase® Reporter Assay kit by Promega. SRF activity was also determined by SRF Luc reporter by the same protocol.

### qPCR

mRNA was isolated by QIAGEN RNeasy Mini kit according to manufacturer protocol and was reversely transcribed to cDNA by a High‐capacity cDNA Reverse Transcription kit (Thermo Fisher). After that, qPCR was performed with GAPDH serving as the housekeeping gene. Primers were purchased from Metabion (Metabion, Planegg, Germany). The sequences of primers used for qPCR analysis are as follows:

CTGF: forward: 5′‐TGGAGTTCAAGTGCCCTGAC‐3′, reverse: 5′‐CTCCCACTGCTCCTAAAGCC‐3′.

CYR 61: forward: 5′‐ACCCTTCTCCACTTGACCAG‐3′, reverse: 5′‐CTTGGCGCAGACCTTACAG‐3′.

GAPDH: forward: 5′‐ACGGGAAGCTTGTCATCAAT‐3′, reverse: 5′‐CATCGCCCCACTTGATTTT‐3′.

RT‐qPCR experiments were performed on an Applied Biosystems® 7300 Real‐Time PCR System (Thermo Fisher, Waltham, MA, USA) with the standard procedures. SYBR™ Green Master Mix (Thermo Fisher) was used for detection of amplified cDNA.

### Formation of spheroids

Spheroids of nontargeting and CDK5 knockdown Huh7 cells were formed overnight using the hanging drop method. Each drop comprised of 1000 cells and total cell density was 50 000 cells·mL^−1^ used with 20% methocel stock solution with cell culture medium. Next day, the spheroids were harvested and resuspended in mixture of collagen solution (rat tail Collagen I from Ibidi, Gräfelfing, Germany) according to the manufacturer's instructions. After 24 h, spheroids were visualised in bright field microscope, and size was determined.

### Immunostaining

All the staining experiments were performed in Ibitreat® 8 well μ‐slides from Ibidi. For immunofluorescence staining, cells were washed with PBS+ and fixed with 4% paraformaldehyde in PBS for 10 min. After washing with PBS, cells were incubated in 0.1% Triton ×‐100 in PBS for 10 min for membrane permeabilization. After another brief washing step, cells were incubated in 1% BSA in PBS for 1 h at room temperature for blocking of unspecific binding sites. After blocking, cells were incubated with primary antibodies diluted in 0.2% BSA in PBS (1 : 200) overnight at 4 °C. The next day, slides were washed 3 × 10 min with 1% BSA in PBS and then incubated in secondary antibody solution (1 : 400) and Hoechst 33342 (1 : 100) for nuclear counter stain in PBS for 1 h at RT. Finally, cells were washed for 2 × 10 min with 1% BSA in PBS and once with PBS for 10 min. Slides were sealed with FluorSave Reagent (Merck, Darmstadt, Germany) and stored at 4 °C in the dark.

### Laser scanning confocal microscopy

Confocal images were acquired with a Leica TCS SP8 SMD confocal microscope, equipped with an HC PL APO 63×/1.40 oil objective and photomultiplier (PMT) or HyD detectors. Pinhole size was adjusted to 1.0 airy units and sequential scanning was performed with a scanning speed of 400 Hz. Following excitation laser lines were applied: 405, 488 and 647 nm.

### Calculation of nucleus‐to‐cytoplasm ratio

For the calculation of the nucleus‐to‐cytoplasm ratio, the nucleus‐to‐cytoplasm intensity tool from fiji was employed (Intensity Ratio Nuclei Cytoplasm Tool, RRID:SCR_018573). For every repetition, 10 images were evaluated, and three repetitions were done. In each image, there were about 70–80 cells which were evaluated by the plugin. Later quantifications were made using the Intensity Ratio Nuclei Cytoplasm Tool plugin and unpaired *t*‐test followed by Welch's correction was employed, and later *P* value was determined.

### Western blot

Cells were washed with ice‐cold PBS 2× times. After that, cold RIPA plus lysis buffer containing inhibitors was added. Cell lysates were stored at −80 °C for at least 1 h or overnight. After thawing, the cells were scraped off with a cell scraper and the resulting residual was transferred to 1.5 mL tubes. After centrifugation (20 000 ***g***, 4 °C, 10 min), the pellet was discarded. Protein concentration was determined by Bradford assay. All samples were diluted to 1 : 10, and absorbance was measured at 592 nm using SpectraFluor Plus™. The final protein concentration was determined using a standard BSA linear standard dilution curve. All the cell lysates were then prepared accordingly by diluting them with 5× sample buffer and were stored at −20 °C until further western blot analysis.

Proteins were separated using SDS/PAGE electrophoresis (80 V, 90 min, RT) and subsequently transferred to nitrocellulose membranes (0.25‐μm pore size) (100 V, 1.5 h, RT). After that, membranes were incubated in blocking solution (5% BSA in TBS‐T and 5% Blotto in TBS‐T) for 2 h at RT followed by incubation in primary antibodies overnight at 4 °C. Subsequently, membranes were washed 3 times with 1× TBS‐T for 5 min each. Then, the membranes were incubated in secondary antibody for 2 h followed by four washing steps with TBS‐T, 5 min each. In some cases, membranes were cropped to allow for simultaneous detection of high molecular weight and low molecular weight proteins (e.g. LATS1 and LATS2 in Fig. 3, the IP experiment in Fig. 6). After that, chemiluminescence was detected by incubating the membranes with ECL solution and 2.5 mm luminol. HRP‐conjugated proteins were detected by ChemiDoc™ Touch Imaging System (Bio‐Rad, Munich, Germany), and the positions of the bands were compared with Page Ruler™ Plus Prestained Protein Ladder. Normalisation of protein loading was performed using the image lab™ Software (Bio‐Rad, Munich, Germany).

### Co‐immunoprecipitation

For Co‐IP experiments, cells were first rinsed 3× with ice‐cold PBS and then precooled RIPA lysis buffer was added. After that, the cells were stored at −80 °C for at least 1 h or overnight. Cells were scraped off with a cell scraper, and the resulting residual was transferred to 1.5 mL tubes. The lysates were recovered after centrifugation, and the protein concentration was determined using the Bradford assay. All steps were performed on ice.

Afterwards, 2 μg of monoclonal antibody and 50 μL of μMACS Protein G MicroBeads were added to the lysates. The lysates were then incubated at 4 °C overnight with shaking. The next day the conjugated protein was recovered by magnetic separation under a strong magnetic field using a magnetic μMACS Column. After incubation with the protein solution, the column was rinsed 3× with RIPA buffer and 1× with low salt buffer. After that, the column was rinsed with preboiled 20 μL SDS/PAGE sample buffer and incubated for 5 min. Later, the protein was eluted in fresh 1.5 mL tubes by applying 50 μL preboiled sample buffer. Eventually, the protein concentration was adjusted by adding SDS/PAGE sample buffer and the samples were boiled for 5 min at 95 °C and stored at −20 °C and then subsequent western blot was done. IgG isotype controls were used as negative control.

### Sample preparation for proteomics

For phosphoproteomics, cells were initially seeded on 6 well dishes (1*10E6 cells per well), trypsinised and thoroughly washed 5× with PBS to remove any residues for FCS and cell culture media. Cells were lysed in 60 μL 8 m Urea/0.4 m NH_4_HCO_3_ using a sonicator (Sonopuls HD3200 + BR‐30, Bandelin, Berlin, Germany), and proteins were reduced by addition of dithioerythritol (6.7 μL of a 50 mm solution in water) and incubation at 37 °C for 30 min. For cysteine carbamidomethylation, iodoacetamide was added (11.8 μL of a 0.1 m solution in water) and incubated for 30 min in the dark at RT. Then, a first digestion step was done using LysC (1 : 100, enzyme : protein, FUJIFILM Wako Chemicals Europe GmbH, Neuss, Germany) at 37 °C for 4 h. Next, the samples were diluted with water to give a concentration of 1 m urea and a second digestion step using modified porcine trypsin (1 : 50 protein to trypsin ratio, Promega) at 37 °C overnight. The reaction was stopped by acidification with formic acid. Phosphopeptide enrichment was carried out with High Select™ Phosphopeptide Enrichment Kits & Reagents (#A32993, Thermo Fisher Scientific) according to the manufacturer protocols.

### Liquid chromatography–mass spectrometry (LC–MS)

For the whole proteome analysis, peptides were separated with an EASY‐Spray column (PepMap RSLC C18, 75 μm × 50 cm, 2 μm particles, Thermo Fisher Scientific) at a flow rate of 250 nL·min^−1^ using a two‐step gradient: Starting with 3% B to 25% B in 160 min, followed by a 10 min ramp to 40% B (A: 0.1% formic acid in water, B: 0.1% formic acid in acetonitrile). For the phosphoproteome analysis, using the same eluents, the gradient started from 3% to 25% B in 30 min, followed by ramping to 40% B in 5 min.

For peptide identification and quantification, an online coupled Q Exactive HF‐X mass spectrometer (Thermo Fisher Scientific) was used, which was run in the data‐dependent acquisition mode with a maximum of 15 MS/MS spectra per survey scan for the analysis of cell lysates. For the phosphoproteome, a maximum of 5 MS/MS spectra was used. Survey scans were measured with a resolution of 60 000 at 200 m/z, and product ion spectra were produced with higher‐energy collisional dissociation (HCD) and analysed with a resolution of 15 000 at 200 m/z.

### Data analysis

Raw spectra files from both the full proteome and phosphoproteome samples were searched with maxquant (v. 2.0.3.0) using Human subset of the Swiss‐Prot and the standard contaminants database. The PTM and match between runs features were turned on. False discovery rate (FDR) was controlled to be 1%. Statistical evaluation and analysis were done using perseus (v. 1.6.5.0) and r. Fold change and *q* values between KD and NT groups were calculated, and volcano plots were generated after performing Welch's *t*‐test and applying a FDR of 5%. Proteins were only considered significantly altered if they had a log2‐fold change larger than 0.6. Phosphosite plus was used for further phosphoproteome analysis.

## Results

### CDK5 interacts with MST2 (STK3)

**Fig. 1:**
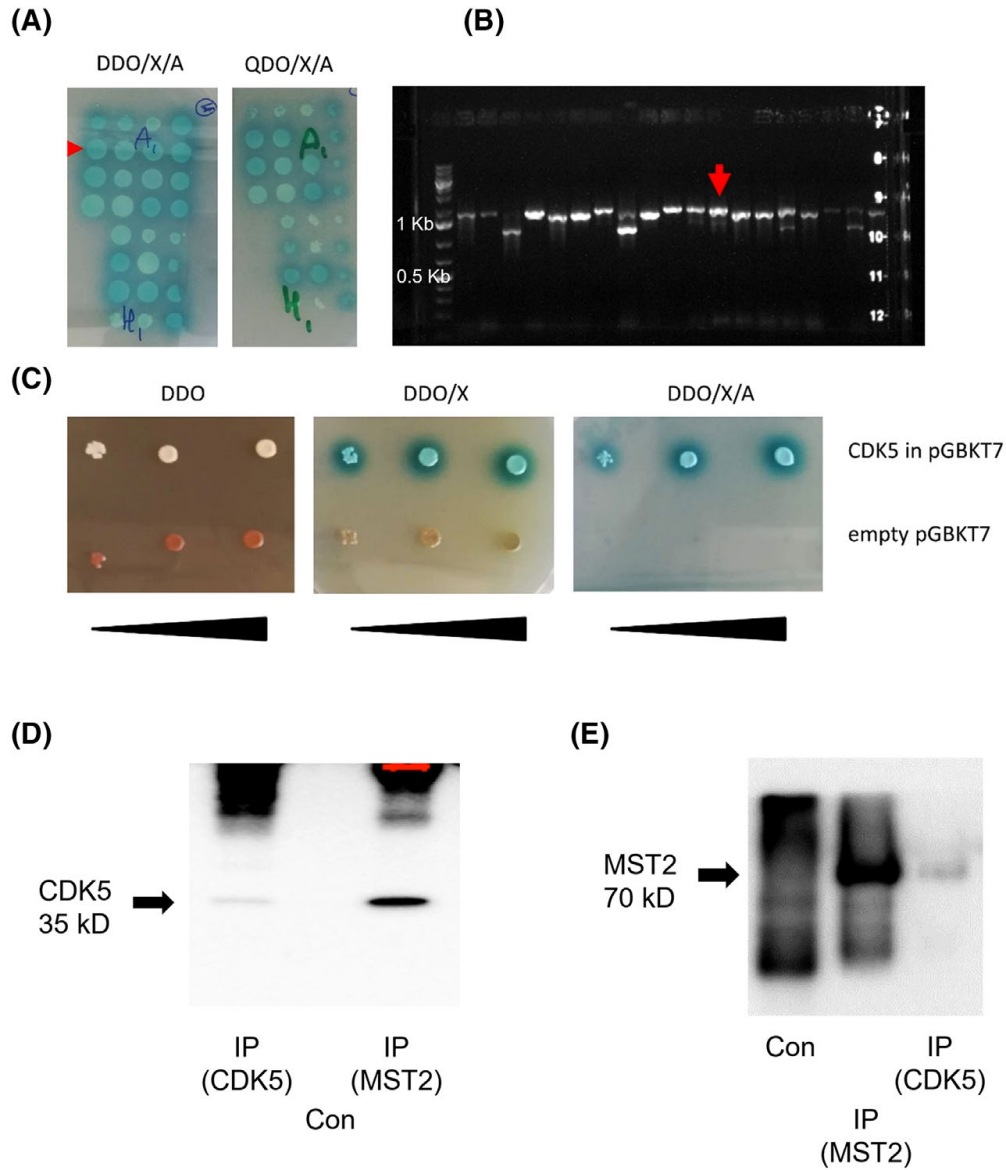
MST2 is an interaction partner of CDK5. (A) Y2H screening of CDK5. The screening was performed using DDO/X/A as selective medium. 316 blue colonies were obtained, which were respotted onto selective plates with increasing stringency (DDO/X/A and QDO/X/A, example shown in panel A). Only colonies, which showed robust growth on both plates, were selected, colony PCR was performed (B), and samples were analysed by Sanger sequencing (size marker for DNA: Gene ruler 1 kb plus, the markers for 0.5 and 1 kb are indicated). Of the 36 colonies containing a plasmid with the insert encoding for MST2, one colony was selected (marked with a red arrow in A and B). The plasmid was isolated, and a prey rescue experiment was performed (C). The prey plasmid with the MST2 insert and the empty prey plasmid were retransformed into the Y2H Gold reporter strain with the CDK5 bait plasmid. A Y2H spot test was performed, in which the two strains (empty prey plasmid and prey plasmid with CDK5 insert) were spotted on DDO plates (confirmation of prey and bait plasmid being present), and two selective plates (DDO/X gives blue colonies if an interaction takes place but the yeast can also grow without the interaction, and DDO/X/A plates, on which the yeast can only grow if an interaction takes place). The prey rescue experiment clearly confirms the interaction between CDK5 and MST2 in the Y2H setup. Black triangles represent serial dilutions. (D and E) Co‐immunoprecipitation experiments confirming this interaction (D) IPs for MST3 and CDK5 were blotted for CDK5 (35 kDa), or (E) for MST2 (STK3). CDK5 was detected in the MST2 IP, and MST2 in the CDK5 IP, which corroborates their interaction. Controls (Con) are IPs with an irrelevant isotype‐matched antibody.

Performing a yeast two‐hybrid screening using the Matchmaker Gold Yeast Two‐Hybrid System (Clontech Takara, San Jose, CA, USA) to detect interaction partners of CDK5, MST2 was determined as one of the major hits and was identified 36 times *i* (Table S1). MST2 was confirmed as hit by a prey rescue experiment (Fig. 1A–C). Interestingly, also cyclin I (CCNI), a previously known interactor of CDK5 [17], was identified in the yeast two‐hybrid screen (Table S1).

Next, to substantiate the interaction between MST2 and CDK5 in an independent approach, we conducted co‐immunoprecipitation experiments in Huh7 cells, in which either a CDK5 antibody was utilised for immunoprecipitation of MST2 and detection via western blot, or *vice versa*. We observed bands of MST2 and CDK5 at 75 and 35 kDa, respectively, confirming the interaction between CDK5 and MST2 (Fig. 1D,E).

### CDK5 knockdown reduces YAP activity

To investigate potential functional consequences of the interaction between MST2 and CDK5, we analysed YAP activity changes after stable CDK5 knockdown. To this end, we performed a YAP reporter gene assay with Huh7 nontargeting (nt) cells and Huh7 CDK5 knockdown (KD) cells.

**Fig. 2:**
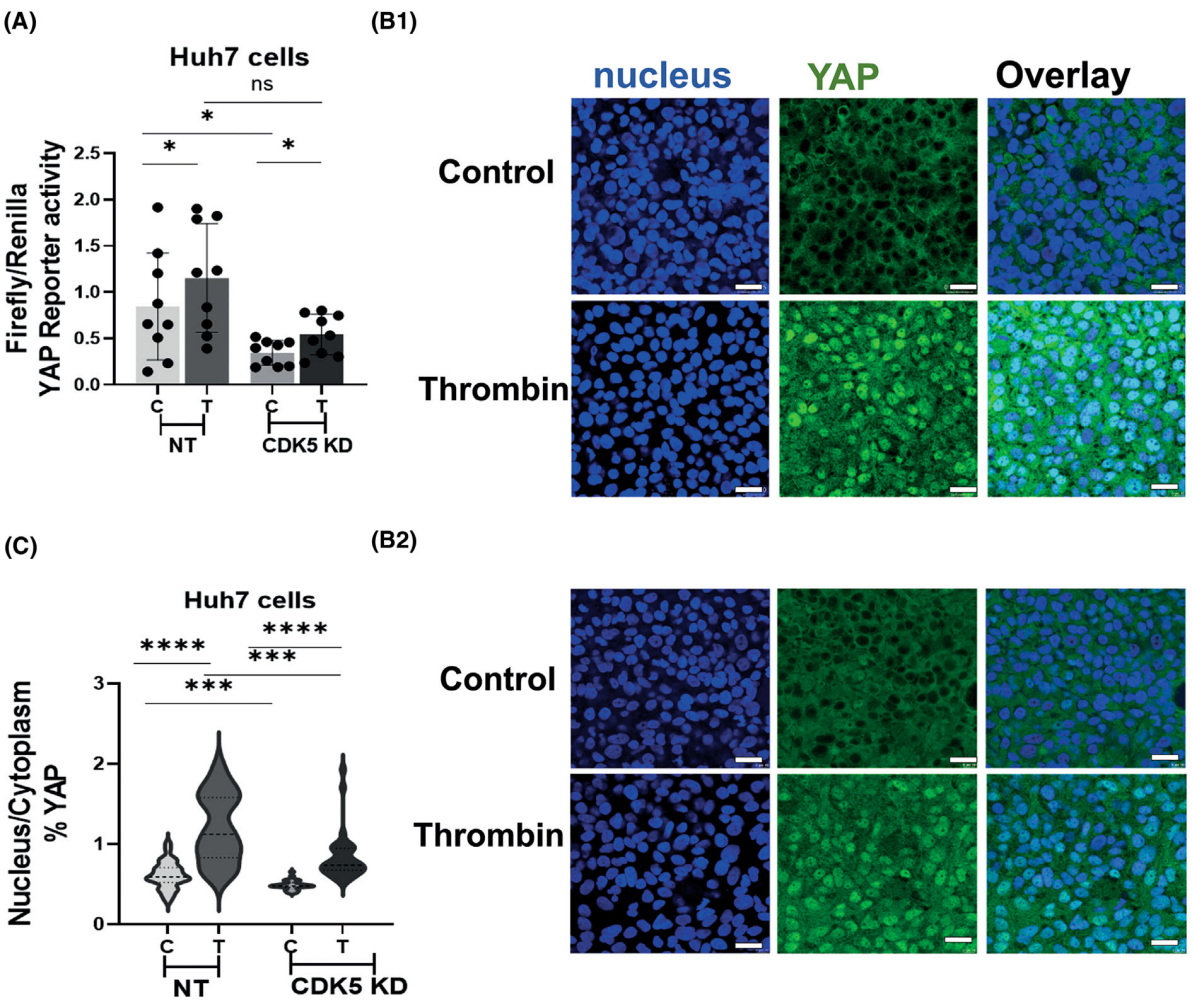
CDK5 knockdown reduces YAP reporter activity. (A) Dual‐luciferase reporter gene assay for TAED 8xGTIIC‐ response element comparing the YAP reporter activity of Huh7 nt controls cells (NT) and Huh7 CDK5 Knockdown clones (CDK5 KD) with (T) and without (C) 0.5 U Thrombin stimulation. Luciferase reporter activity is expressed as firefly RLU normalised to the constitutive renilla control construct (mean ± SEM for three independent experiments, unpaired *t*‐test followed by Welch's correction, *P < 0.05, ns, not significant). (B) Immunostaining of YAP on Huh7 nt control cells (panel B1) and Huh7 CDK5 knockdown clones (panel B2) seeded on plastic dishes with high cell density, 2*10E5 cells per well, with and without thrombin stimulation, (0.5 U, 30 min). (C) Nucleus‐to‐cytoplasm YAP intensity ratio analysed with the Intensity Ratio Nuclei Cytoplasm Tool plugin for fiji imagej software and presented as super graph (mean ± SEM for three independent experiments with 10 images per experiment, unpaired *t*‐test followed by Welch's correction, ***P < 0.05, ****P < 0.0001). Scale bars: 10 μm.

Interestingly, we found that Huh7 CDK5 KD clones have reduced YAP reporter activity (almost by half), as compared to Huh7 nt control cells (Fig. 2A). We treated the cells with thrombin for 30 min to stimulate and activate YAP. Upon thrombin treatment, reporter activity of YAP increased in both Huh7 CDK5 KD clones and Huh7 nt control cells. However, the reporter activity is on a lower level irrespective of stimulation after CDK5 knockdown (Fig. 2A). Since it has been widely accepted that cell confluence regulates YAP nucleocytoplasmic shuttling [18, 19, 20], we performed all experiments with Huh7nt and Huh7 CDK5 KD cells at high cell density. Investigation of YAP localisation by immunofluorescence staining clearly showed the nuclear translocation of YAP into the nucleus upon treatment with thrombin. In contrast to the reporter gene assay, however, activation by thrombin in terms of nuclear to cytoplasmic ratio was reduced in the CDK5 KD group as compared to nt (Fig. 2B,C).

In a next step, we investigated transcriptional regulation of two YAP‐target genes, CTGF [21] and CYR61 [22] by qPCR. To our surprise, knockdown of CDK5 did not cause a reduction of mRNA for CTGF, and even increased transcription of CYR61 (Fig. S2). Since there is an intense crosstalk between YAP‐ and SRF/MRTF signalling, and since CTGF and CYR61 also are SRF‐target genes [23], we also monitored SRF activity with a respective reporter gene assay. Interestingly, SRF reporter activity was upregulated in CDK5 knockdown cells (Fig. S3), which might indicate a compensatory response to YAP inhibition.

### Knockdown of CDK5 reduces spheroid growth

Since YAP is involved in growth of tumour cell spheroids in 3D models [24], we investigated spheroid size in nontargeting and CDK5 knockdown Huh7 cells. Indeed, spheroid size decreased in the CDK5 knockdown cells (Fig. S4). This has, however, to be interpreted with care, since CDK5 has pleiotropic activity, and might contribute to spheroid size regulation in other ways also.

### CDK5 influences the phosphorylation levels of kinases involved in the Hippo pathway

**Fig. 3:**
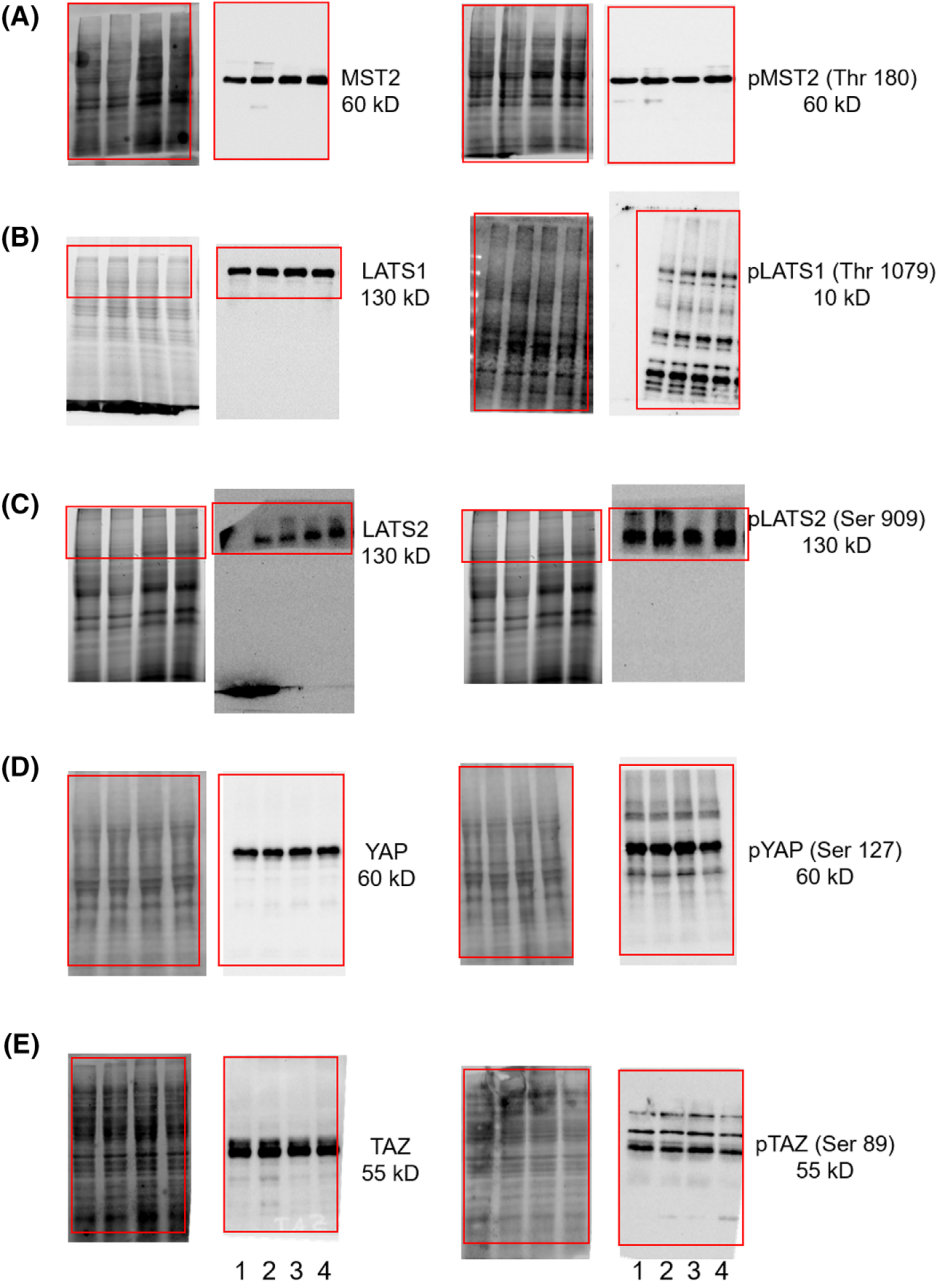
Influence of CDK5 on the phosphorylation levels of kinases involved in the Hippo pathway. (A–E) Representative western blots of, Huh7 nt (NT) and Huh7 CDK5 knockdown cells (CDK KD) for total and phosphorylated levels of upstream Hippo pathway kinases (A) MST2 and pMST1 (Thr 180). pMST (Thr 183), (B) LATS1 and LATS1 (Thr 1079), (C) LATS2 and LATS (Ser 909) and downstream factors (D) YAP and pYAP (Ser 127) (E) TAZ and pTAZ (Ser 89) with (T) and without (C) thrombin treatment (0.5 U, 15 min treatment for upstream and 1 h treatment for downstream factors), left panels show the loading controls for the respective protein or phosphoprotein, the positions of the loading controls and the stained membranes are matched as indicated by the red frames. Please note that the membranes for LATS1 and LATS2 were cut before antibody incubation (the lower molecular weight parts were used for different proteins). In cases where phosphoproteins show multiple bands, the one matching in molecular weight to the band of the total protein was used for quantification. Lane 1: Control cells, lane 2: control cells after thrombin stimulation, lane 3: untreated CDK5 knockdown cells, lane 4: CDK5 knockdown cells after stimulation with thrombin. Representative images from *n* = 3 experiments.

**Fig. 4:**
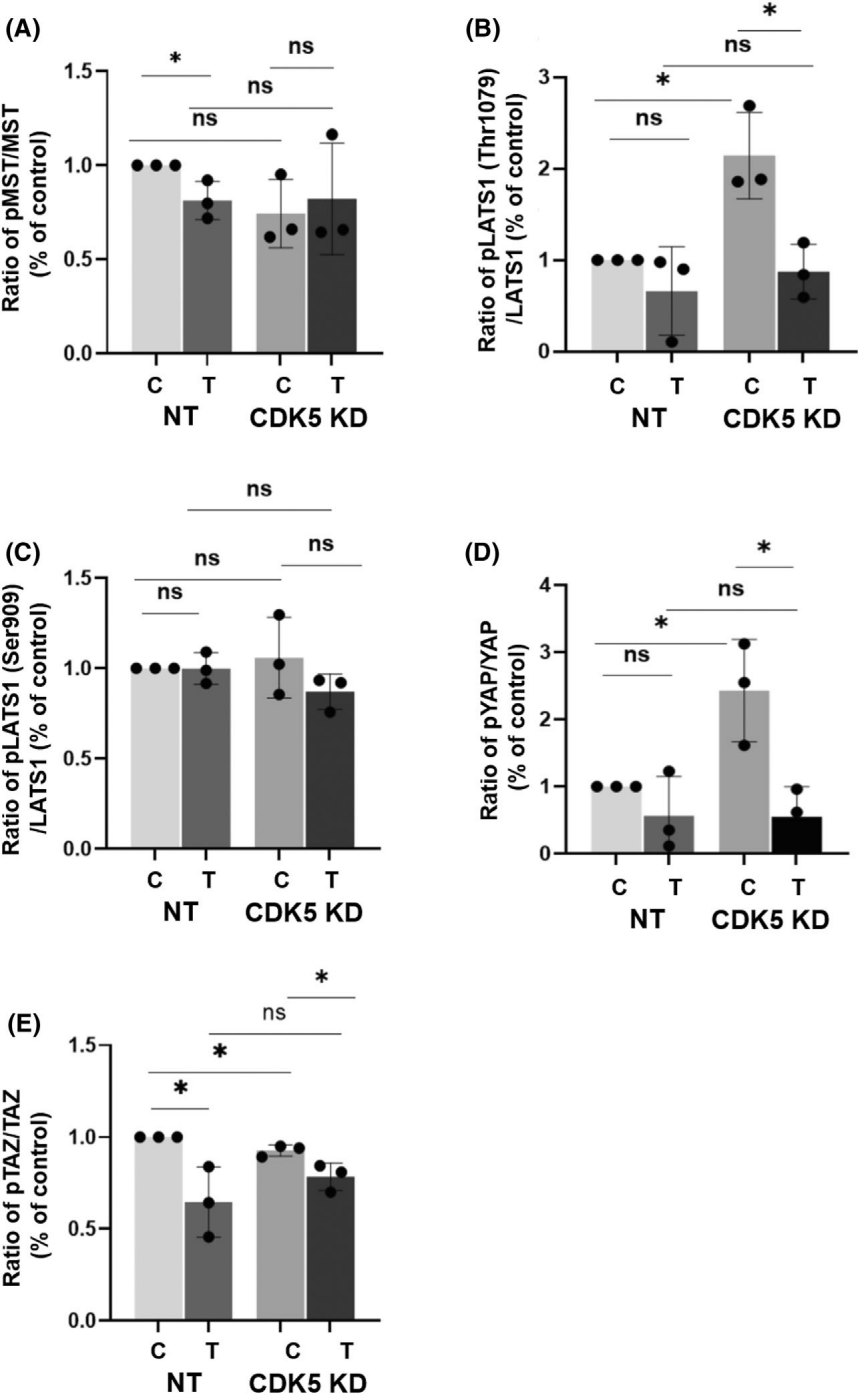
Influence of CDK5 on the phosphorylation levels of kinases involved in the Hippo pathway. (A–E) Ratio of phosphorylated protein levels to total protein levels normalised to the loading control band intensity (Huh7 nt control cells). In cases where phosphoproteins show multiple bands, the one matching in molecular weight to the band of the total protein was used for quantification. The experiments are performed in triplicates and bar graphs indicate ± SEM, one‐way ANOVA test followed by Dunnett's multiple comparison test, ANOVA overall summary, *P < 0.05, ns, not significant.

Next, since CDK5 is a kinase, we investigated the phosphorylation status of upstream Hippo pathway kinases and downstream effectors with and without stimulation with thrombin (Fig. 3). The expression levels of the examined proteins did not vary between experimental groups (Fig. 3A–E). The level of phospho MST1/MST2 (ratio of phospho levels of protein to total protein) remained almost the same in all groups (Figs 3A and 4A). Interestingly, however, there was a significant increase in phospho LATS1 (Thr 1079) in the case of the CDK5 knockdown (Figs 3B and 4B) both with and without stimulation with thrombin. However, a different phosphorylation site (Ser 909) of LATS was not affected (Figs 3C and 4C).

Downstream, the total expression of the oncoproteins YAP and TAZ did not vary at all in the experimental groups (Fig. 3D,E, left panels). Nevertheless, the phospho levels of YAP in the case of CDK5 knockdown were higher (Figs 3D and 4D) and dropped upon treatment with thrombin in both cases. The levels of phospho TAZ in the case of CDK5 knockdown did not vary much (Fig. 3E). However, upon thrombin treatment, the phospho level of TAZ was reduced in both groups (Fig. 4E).

### The kinase activity of CDK5 does not influence phospho YAP levels but the localisation of YAP in the nucleus

**Fig. 5:**
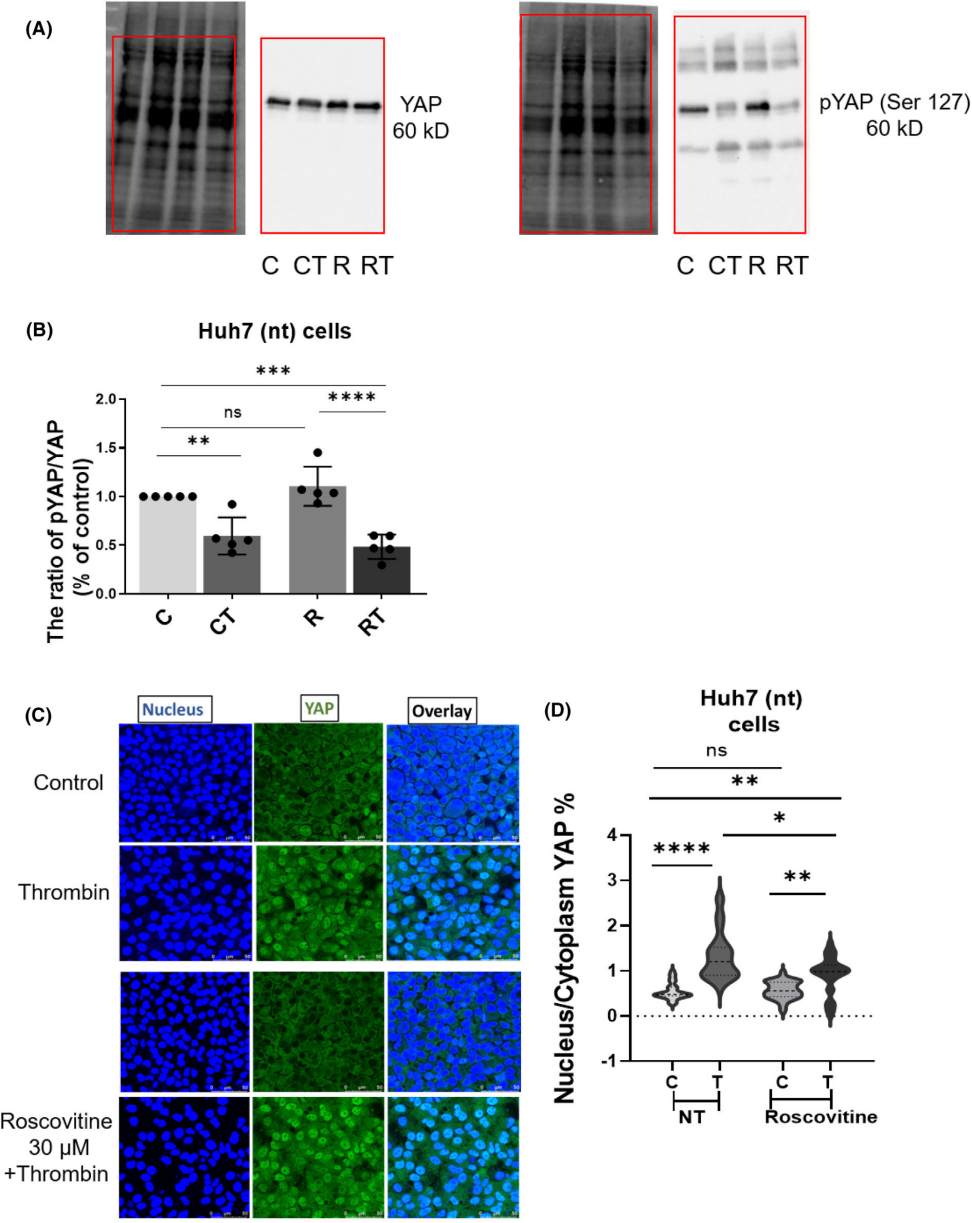
CDK5 does not influence phospho YAP levels but to some degree localisation of YAP in the nucleus. To determine the effect of pharmacological inhibition of the kinase activity of CDK5, cells were treated with roscovitine, (A) western blot for YAP (left panel) and pYAP (right panel) on Huh7 nt cells (C, untreated control cells; CT, cells with thrombin treatment; R, cells with roscovitine treatment; RT, cells treated with roscovitine followed by thrombin stimulation). The cells were treated with roscovitine (50 μm, 3 h) followed by thrombin treatment (0.5 U, 1 h) as indicated. The positions of the loading controls and the stained membranes are matched as indicated by the red frames. (B) Ratios of phosphorylated YAP and total YAP in the four groups. All values were normalised to the respective control band intensities. The experiments were performed in triplicates, and bar graphs indicate mean ± SEM (one‐way ANOVA followed by Turkey's multiple comparison test). (C) Immunostaining for YAP in all four groups. For these experiments, cells were treated with roscovitine (30 μm, 3 h) followed by a thrombin stimulation (0.5 U, 30 min). (D) Calculation of nucleus‐to‐cytoplasm intensity ratio for YAP (the experiment was conducted in triplicates and for every repetition 10 images were taken into account, values are presented as mean ± SEM, unpaired *t*‐test followed by Welch's correction).

We next investigated whether the kinase activity of CDK5 is a prerequisite for regulating the levels of pYAP (Ser 127) by using the well‐established CDK5 inhibitor roscovitine. Huh7 nt cells were treated with 50 μm roscovitine for 3 h followed by thrombin treatment for 1 h. Then, YAP and pYAP levels were determined, and the pYAP/total YAP ratio was calculated by normalising the values to control (Fig. 5A,B). Interestingly, there was no significant change in the levels of phosphorylated YAP in roscovitine‐treated cells compared to control cells (Fig. 5A,B).

Next, we did immunostaining experiments to look for YAP localisation after roscovitine treatment and then calculated nucleus‐to‐cytoplasm intensity (Fig. 5C,D). For this, cells were treated with 30 μm roscovitine for 30 min and then later with thrombin for 30 min. Compared to the untreated control, thrombin caused nuclear translocation of YAP irrespective of the pretreatment with roscovitine, however, to a significantly lesser degree after treatment with roscovitine. To some degree, phosphorylation and translocation of YAP might be divergent after inhibition of CDK5 kinase activity. Inhibition of the kinase activity of CDK5 thus leads to effects different from silencing of CDK5 and might hint towards a different mode of action of CDK5, for example as binding partner in protein–protein binding or formation of ternary complexes.

### CDK5 knockdown and changes in the proteome and phosphoproteome

Since we had ruled out direct phosphorylation of components of the Hippo pathway by CDK5, we performed a comparative proteome and phosphoproteome analysis in Huh7 nt and CDK5 KD cells to investigate a potential connection between Hippo and CDK5 signalling in an unbiased way. A total number of about 3300 proteins were identified in the whole proteome analysis. The principal component analysis (PCA) of protein intensity values showed a clear separation between genotypes, demonstrating that CDK5 KD induces major effects on the cellular Huh7 proteome (Fig. S5). Statistical evaluation led to the detection of 583 more and 327 less abundant significant proteins in CKD5 KD cells.

**Fig. 6:**
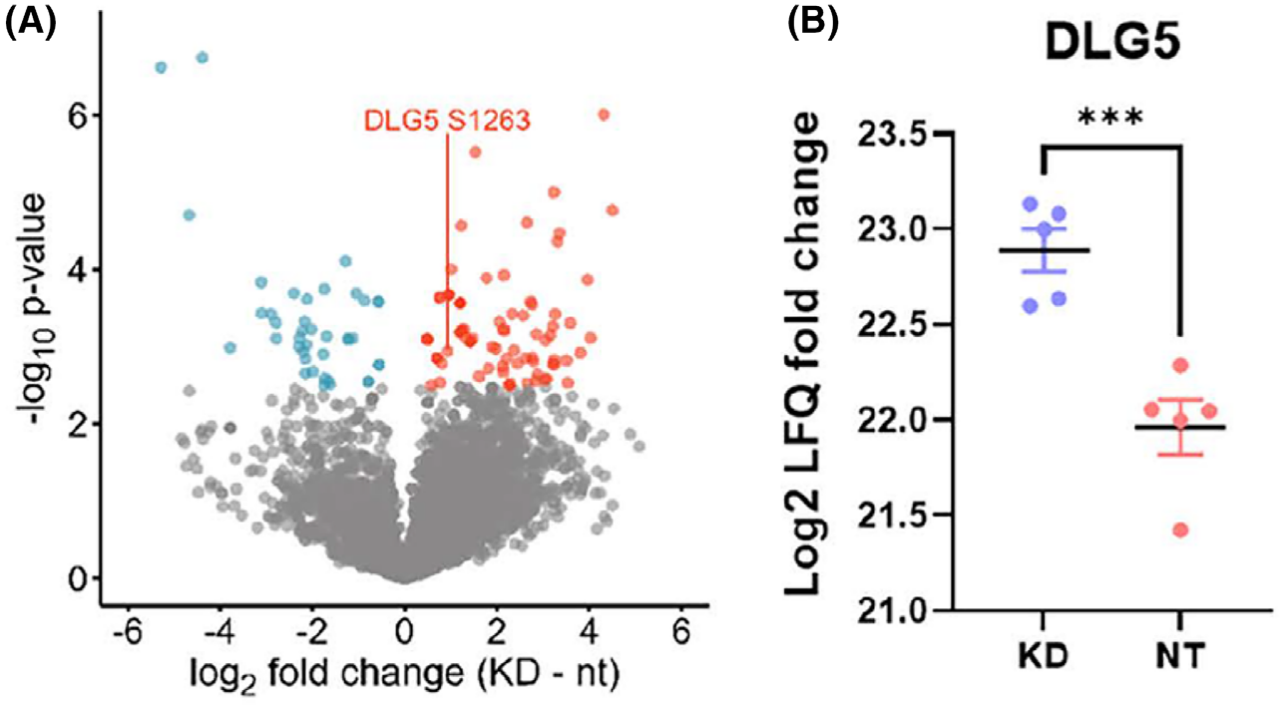
Knockdown of CDK5 influences the phosphorylation of proteins involved in Hippo signalling. (A) Volcano plot determining the changes in the phosphorylation status of the proteins in Huh7 CDK5 KD and Huh7 nt cells with red dots representing higher phosphorylation in KD, blue dots representing reduced phosphorylation in KD, and grey no significant change. (B) Differences in the phosphorylation intensity of DLG5 represented in terms of Log2 LFQ change, LFQ represents label free quantified values from Mass spectroscopy analysis (*n* = 3, ***P < 0.001). Values are presented as mean ± SEM, unpaired *t*‐test followed by Welch's correction.

For phosphoproteome analysis, only peptides with phosphosite localisation possibilities of > 0.75 and score difference of > 5 were included, resulting in identification of about 5000 phosphosites. Statistical analysis (Welch's *t*‐test with FDR < 5%), revealed 123 phosphopeptides significantly differing in abundance between Huh7 nt and CDK5 KD cells (Fig. 6A). Notably, there were several established interacting partners (based on the protein–protein interaction database BioGRID) of YAP1, MST2 and CDK5 that were differentially phosphorylated upon knockdown of CDK5, indicating a crosstalk between these pathways, as displayed in Tables S2–S4. In search of a link between CDK5 and Hippo signalling, we focussed on disk large homologue 5 (DLG5), a membrane associated guanylate kinase (MAGUK) protein, which plays a role in cell polarity, and which is a known interactor of MST2 and regulator of the Hippo pathway [25]. Phosphorylation of DLG5 at S1263 is increased upon knockdown of CDK5 (Fig. 6A,B).

## Discussion

We identified a novel interaction between MST2 (STK3) and CDK5. MST1/2 acts as an upstream regulator of the Hippo pathway and regulates the expression of the oncoproteins YAP and TAZ by phosphorylating LATS1/LATS2. LATS1/LATS2 are independently phosphorylated also by MAP4K kinases [26]. We demonstrate that following the interaction with MST2, CDK5 modulates the Hippo pathway and contributes to the activity of the oncoprotein YAP by tempering the levels of phosphorylated YAP. Knockdown of CDK5 increases the levels of phosphorylated upstream kinases LATS1 (Thr 1079), which in turn rises phosphorylation levels of YAP, and results in reduced YAP activity. This is in good accordance with recent studies, which suggest that CDK5 attenuates the Hippo pathway in melanoma and lung cancer [27, 28]. However, in these studies no mode of action of CDK5 in this context has been proposed. Intriguingly, CDK5 seems to interact with MST2, although no CDK5‐specific phosphorylation was observed.

CDK5 is a major kinase and has a wide variety of substrates, which it phosphorylates in many functional settings. Apart from CNS development, all these substrates play an essential role in the regulation of cytoskeletal dynamics, pro‐ and antimigratory processes, synaptic functions and membrane cycling [29, 30]. However, our results suggest that pharmacological inhibition of kinase activity of CDK5 by roscovitine [31] has no prominent effect on the phosphorylation status of YAP, while knockdown of CDK5 does. This could mean that it is not essentially the kinase activity of CDK5 that is responsible for the modulation of the Hippo cascade, but that it could act as a part of a protein scaffold. It might also be speculated that the interaction of MST2 with CDK5 might regulate signalling independent of the Hippo core.

Due to its many functions, knockdown of CDK5 influences the entire proteome and phosphoproteome. Interestingly, we found significant changes in the phospho levels of important YAP, MST2 and CDK5 interacting partners upon knockdown of CDK5 (Tables S2–S4). DLG5 is one such interacting partner of MST2 belonging to this category of interactors [32]. It has been well established that DLG5 directly regulates the Hippo pathway by promoting hyperphosphorylation of MST2 through recruitment of microtubule affinity regulating kinase 3 (MARK3) to MST2. DLG5 also assists in interaction of MST2 and LATS1 [33]. Since phosphorylation of DLG5 increased upon knockdown of CDK5, DLG5 is obviously no direct substrate of CDK5, although they seem to be functionally related. CDK5 might be part of a protein complex with MST2, thus regulating the interactions between MST2 and DLG5, which modulate YAP activity.

Our study adds a new dimension to the Hippo pathway regulation. We provide new insights into how the interaction between CDK5‐MST2 results in the transcriptional regulation of the Hippo pathway in Huh7 cells, which could be a new therapeutic strategy for the development of CDK5 modulators to target the dysregulated Hippo pathway.

## Conflict of interest

The authors declare no conflict of interest.

### Peer review

The peer review history for this article is available at https://www.webofscience.com/api/gateway/wos/peer‐review/10.1002/2211‐5463.13962.

## Author contributions

MP performed experiments, analysed data; JBS performed experiments, analysed data; TF analysed data, supervised; SM performed experiments, data analysis, supervision; AMV supervision, editing of the manuscript, project administration; SZ conceptualized the work, supervision, writing of the manuscript.

## Supporting information


**Fig. S1.** CDK5 is downregulated in the knockdown cell line.
**Fig. S2.** Expression of the YAP target genes CTGF and CYR61 as measured by qPCR is not reduced in CDK5 knockdown cells as compared to non‐targeting cells.
**Fig. S3.** The transcriptional activity of a SRF/MRTF reporter gene as measured in a dual luciferase reporter gene assay is increased after knockdown of CDK5.
**Fig. S4.** Representative images of spheroid growth of non‐targeting and CDK5 knockdown cells in a collagen gel.
**Fig. S5.** (A) Volcano plot of proteins significantly altered in abundance after knockdown of CDK5 (red: upregulated in KD, blue: downregulated in KD, cutoff for colour coded proteins: *q* value < 0.05, fold change 0.6). (B) Principal component analysis of proteome profiles showing a clear separation of the KD and the NT samples, respectively.
**Table S1.** Sequencing results from the yeast two hybrid system.
**Table S2.** Significantly changed phosphorylation status of known interactors of CDK5 after CDK5 knockdown with Log2 fold change representing changes in LFQ intensity values.
**Table S3.** Significantly changed phosphorylation status of known interactors of YAP after CDK5 knockdown with Log2 fold change representing changes in LFQ intensity values.
**Table S4.** Significantly changed phosphorylation status of known interactors of MST2 after CDK5 knockdown with Log2 fold change representing changes in LFQ intensity values.

## Data Availability

Data will be shared upon reasonable request. Please contact Prof SZ (stefan.zahler@cup.uni-muenchen.de).
