# Supplementary material for: CDK5 interacts with MST2 and modulates the Hippo signalling pathway
Source: FEBS Open Bio. 2024 Dec 30;15(4):647–60. doi: 10.1002/2211-5463.13962 (PMC11961382; doi:10.1002/2211-5463.13962)
Supplement: Supplementary file 1 — Fig. S1. CDK5 is downregulated in the knockdown cell line. Fig. S2. Expression of the YAP target genes CTGF and CYR61 as measured by qPCR is not reduced in CDK5 knockdown cells as compared to non‐targeting cells. Fig. S3. The transcriptional activity of a SRF/MRTF reporter gene as measured in a dual luciferase reporter gene assay is increased after knockdown of CDK5. Fig. S4. Representative images of spheroid growth of non‐targeting and CDK5 knockdown cells in a collagen gel. Fig. S5. (A) Volcano plot of proteins significantly altered in abundance after knockdown of CDK5 (red: upregulated in KD, blue: downregulated in KD, cutoff for colour coded proteins: q value < 0.05, fold change 0.6). (B) Principal component analysis of proteome profiles showing a clear separation of the KD and the NT samples, respectively. Table S1. Sequencing results from the yeast two hybrid system. Table S2. Significantly changed phosphorylation status of known interactors of CDK5 after CDK5 knockdown with Log2 fold change representing changes in LFQ intensity values. Table S3. Significantly changed phosphorylation status of known interactors of YAP after CDK5 knockdown with Log2 fold change representing changes in LFQ intensity values. Table S4. Significantly changed phosphorylation status of known interactors of MST2 after CDK5 knockdown with Log2 fold change representing changes in LFQ intensity values. [file FEB4-15-647-s001.pdf]

CDK5 interacts with MST2 and modulates the Hippo signalling pathway

Mehak Passi, Jan B. Stöckl, Thomas Fröhlich, Simone Moser, Angelika Vollmar and Stefan Zahler

Insert identity	frequency
**Homo sapiens ring finger protein 2 (RNF2)**	25
**Homo sapiens leucine rich repeat containing 6 (LRRC6)**	1
**Cyclin I (CCNI)**	60
**Phosphyglucomutase 1**	1
**Homo sapiens pyridoxal dependent decarboxylase domain containing 2, pseudogene (PDXDC2P) on chromosome16**	21
**Homo sapiens serine/threonine kinase 3 (STK3)**	36

Supplementary Table 1 Sequencing results from the yeast two hybrid system. The identity of the prey plasmid insert was evaluated from the sequencing result using a BLAST search. Frequency refers to the number of colonies from which the according hit was isolated.

Protein names	Gene names	p-value	q-value	Log2 fold change	Amino Acid	Multiplicity	UniProt Accession
Activated RNA polymerase II transcriptional coactivator p15	SUB1	1.65E-03	0.040	0.808	S118	1	P53999
Death-associated protein 1	DAP	2.99E-06	0.052	1.526	S3	1	P51397
Fatty acid synthase [Acyl-carrier-protein] S-acetyltransferase	FASN	7.35E-04	0.040	-1.689	T2204	1	P49327
High mobility group protein HMG-I/HMG-Y	HMGA1	8.56E-04	0.036	1.419	S99	3	P17096
High mobility group protein HMG-I/HMG-Y	HMGA1	8.56E-04	0.035	1.419	S102	3	P17096
High mobility group protein HMG-I/HMG-Y	HMGA1	8.56E-04	0.035	1.419	S103	3	P17096
Nestin	NES	9.71E-07	0.041	4.308	S1496	1	P48681
Microtubule-associated protein 1B;MAP1B heavy chain;MAP1 light chain LC1	MAP1B	2.81E-03	0.050	-0.787	S1785	2	P46821
Microtubule-associated protein 1B;MAP1B heavy chain;MAP1 light chain LC1	MAP1B	2.81E-03	0.050	-0.787	T1788	2	P46821

Supplementary Table 2 Significantly changed phosphorylation status of known interactors of CDK5 after CDK5 knockdown with Log2 fold change representing changes in LFQ intensity values.

Protein names	Gene names	p-value	q-value	Log2 fold change	Amino Acid	Multiplicity	UniProt Accession
Apoptotic chromatin condensation inducer in the nucleus	ACIN1	0.001	0.041	1.229	S208	2	Q9UKV3
Apoptotic chromatin condensation inducer in the nucleus	ACIN1	0.001	0.042	1.229	S216	2	Q9UKV3
Fatty acid synthase; [Acyl-carrier-protein] S-acetyltransferase; [Acyl-carrier-protein] S-malonyltransferase	FASN	0.001	0.040	-1.689	T2204	1	P49327
Heterogeneous nuclear ribonucleoprotein A1;Heterogeneous nuclear ribonucleoprotein A1, N-terminally processed	HNRNPA1	0.000	0.075	-2.402	S368	1	P09651
Microtubule-associated protein 4	MAP4	0.000	0.086	1.771	S696	1	P27816
Nucleolin	NCL	0.000	0.092	2.146	S563	1	P19338
Serine/arginine repetitive matrix protein 2	SRRM2	0.001	0.040	2.767	S353	1	Q9UQ35
Serine/arginine repetitive matrix protein 2	SRRM2	0.000	0.072	-4.384	S1987	1	Q9UQ35
Serine/arginine repetitive matrix protein 2	SRRM2	0.001	0.037	-1.236	S1102	2	Q9UQ35
Serine/arginine repetitive matrix protein 2	SRRM2	0.001	0.037	-1.236	S1103	2	Q9UQ35
Serine/arginine repetitive matrix protein 2	SRRM2	0.000	0.062	0.756	S2692	2	Q9UQ35
Serine/arginine repetitive matrix protein 2	SRRM2	0.000	0.065	0.756	S2694	2	Q9UQ35
Serine/arginine repetitive matrix protein 2	SRRM2	0.002	0.044	-2.151	S1987	2	Q9UQ35
Serine/arginine repetitive matrix protein 2	SRRM2	0.000	0.048	1.200	S968	3	Q9UQ35
Serine/arginine repetitive matrix protein 2	SRRM2	0.000	0.047	1.200	S970	3	Q9UQ35
Serine/arginine repetitive matrix protein 2	SRRM2	0.000	0.050	1.200	S972	3	Q9UQ35
Serine/arginine repetitive matrix protein 2	SRRM2	0.000	0.051	1.200	S973	3	Q9UQ35
Serine/arginine repetitive matrix protein 2	SRRM2	0.000	0.053	1.200	S974	3	Q9UQ35
Serine/arginine repetitive matrix protein 2	SRRM2	0.001	0.035	0.485	S2100	3	Q9UQ35
Serine/arginine repetitive matrix protein 2	SRRM2	0.001	0.036	0.485	S2102	3	Q9UQ35
Serine/arginine repetitive matrix protein 2	SRRM2	0.001	0.037	3.802	S295	3	Q9UQ35
Serine/arginine repetitive matrix protein 2	SRRM2	0.002	0.039	3.500	S300	3	Q9UQ35
Serine/arginine repetitive matrix protein 2	SRRM2	0.001	0.036	0.485	T2104	3	Q9UQ35
Tight junction protein ZO-2	TJP2	0.001	0.043	2.133	S244	1	Q9UDY2
Tight junction protein ZO-2	TJP2	0.000	0.083	3.312	S1067	2	Q9UDY2
Tight junction protein ZO-2	TJP2	0.001	0.039	3.151	S1068	2	Q9UDY2

Ubiquitin-associated protein 2-like	UBAP2L	0.000	0.090	0.998	S609	2	Q14157
Vimentin	VIM	0.002	0.043	2.135	S459	1	P08670
Vimentin	VIM	0.001	0.038	1.469	S51	2	P08670
Vimentin	VIM	0.000	0.077	1.221	S56	2	P08670

Supplementary Table 3 Significantly changed phosphorylation status of known interactors of YAP after CDK5 knockdown with Log2 fold change representing changes in LFQ intensity values.

Protein names	Gene names	p-value	q-value	Log2 fold change	Amino Acid	Multiplicity	UniProt Accession
Disks large homolog 5	DLG5	0,001	0,037	0,927	S1263	1	Q8TDM6
Microtubule-associated protein 4	MAP4	0,000	0,086	1,771	S696	1	P27816

Supplementary Table 4 Significantly changed phosphorylation status of known interactors of MST2 after CDK5 knockdown with Log2 fold change representing changes in LFQ intensity values.

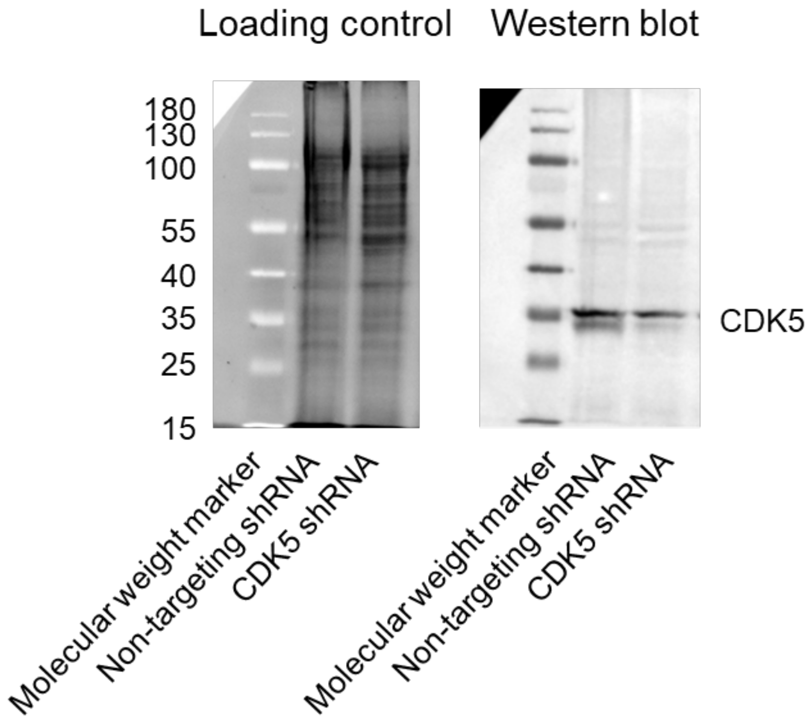

Supplementary Figure 1 CDK5 is downregulated in the knockdown cell line. Left panel: loading control, right panel: Western blot for CDK5. Densitometric analysis after normalization to loading control showed a downregulation to 30% of CDK5 as compared to controls.

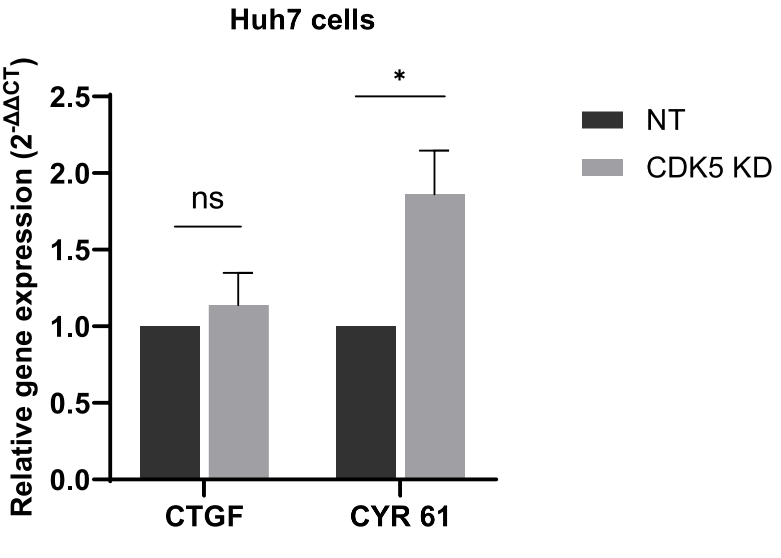

Supplementary Figure 2 Expression of the YAP target genes CTGF and CYR61 as measured by qPCR is not reduced in CDK5 knockdown cells as compared to non-targeting cells (*p < 0.05, ns: not significant, unpaired t-test, data are mean +/- SEM, n = 3).

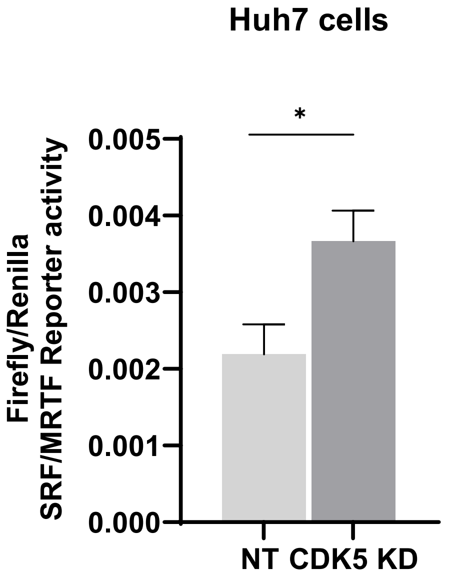

Supplementary Figure 3 The transcriptional activity of a SRF/MRTF reporter gene as measured in a dual luciferase reporter gene assay is increased after knockdown of CDK5. This might be compensatory for a loss of YAP activity. (*$p < 0.05$, unpaired t-test, data are mean +/- SEM, n = 3).

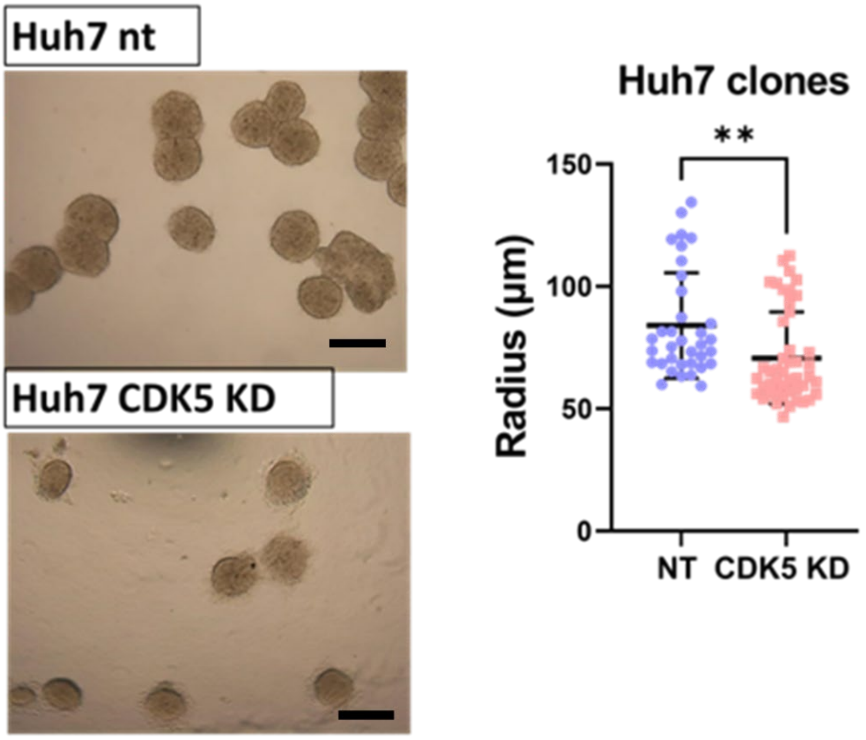

Supplementary Figure 4 Representative images of spheroid growth of non-targeting and CDK5 knockdown cells in a collagen gel (left panels). The size of the spheroids in the CDK5

knockdown cells is significantly reduced in the CDK5 knockdown cells (right panel). (**p < 0.01, unpaired t-test, data are mean +/- SEM, n = 3). Scale bar: 100 µm.

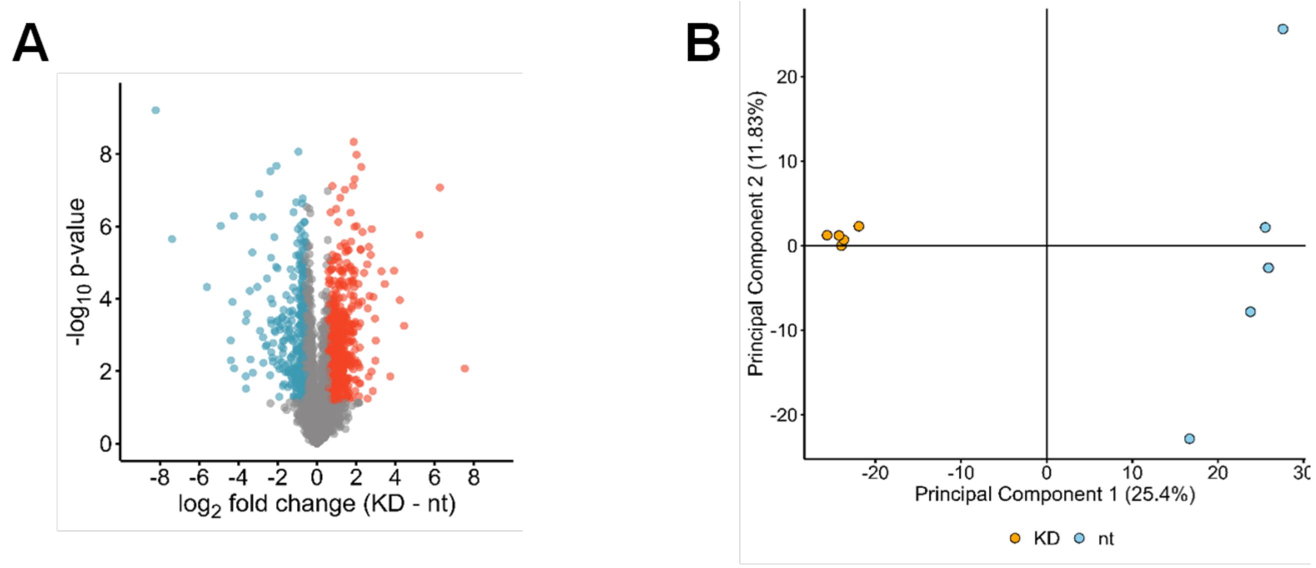

Supplementary Figure 5 A) Volcano plot of proteins significantly altered in abundance after knockdown of CDK5 (red: upregulated in KD, blue: downregulated in KD, cutoff for colour coded proteins: q value < 0.05, fold change 0.6). B) Principal component analysis of proteome profiles showing a clear separation of the KD and the NT samples, respectively.
